# Knowledge of Narcolepsy Among Physicians in Makkah Region, Saudi Arabia: A Cross-Sectional Study

**DOI:** 10.7759/cureus.66052

**Published:** 2024-08-03

**Authors:** Maha K Almatrafi, Dai O Zafer, Reem M Alkhaldi, Tasneem M Moglan, Safa H Alkalash

**Affiliations:** 1 College of Medicine, Umm Al-Qura University, Makkah, SAU; 2 Community Medicine and Health Care, Umm Al-Qura University, Al-Qunfudah, SAU; 3 Family Medicine, Menoufia University, Shebin Elkom, EGY

**Keywords:** cataplexy, sleep medicine, neurology, sleep disorders, narcolepsy

## Abstract

Background

Narcolepsy is a chronic sleep disorder that is characterized by excessive daytime sleepiness and cataplexy. It has been increasingly diagnosed over the years, impacting productivity and employment rates. Awareness of healthcare providers is crucial for the early identification and management of this condition.

Objectives

This study assessed physicians’ knowledge of narcolepsy in the Makkah region of Saudi Arabia.

Method

This cross-sectional study was conducted from February to November 2023. An online self-administered questionnaire has been used to target physicians working in the Makkah region of Saudi Arabia. The utilized questionnaire assessed demographic and professional data as well as the participants’ knowledge of narcolepsy. Statistical analysis was performed using RStudio (R version 4.3.1.). Statistical differences in knowledge were assessed using Pearson’s chi-squared and Fisher’s exact tests. Factors associated with knowledge of narcolepsy were investigated through univariable and multivariable regression analyses expressed using beta coefficients and 95% confidence intervals (95% CIs). Statistical significance was considered at p < 0.05.

Results

A total of 226 physicians participated in this study. Male physicians (54.4%, n = 123), practicing in governmental hospitals (77.9%, n = 176) and residing in Makkah City (43.4%, n = 98) were predominant. Non-surgical specialties represented 73.5% (n = 166) of the sample. Around 81% (n = 184) of the participants were aware of narcolepsy, with a significant difference according to professional status (p = 0.045). The majority of physicians have correctly identified narcolepsy as a sleep disorder (78.3%, n = 177), but only 32.3% (n = 73) have identified its typical onset age group and recognized that there are two types of narcolepsy. Almost half of the respondents indicated a lack of knowledge about the diagnostic criteria for narcolepsy in the DSM-5 (52.2%, n = 118). Only 27.4% (n = 62) recognized the correct diagnostic criteria. Half of the sample (51.3%, n = 116) recognized the use of multiple sleep latency tests for the diagnosis. For factors associated with higher participants’ knowledge, non-surgical specialties showed significantly higher knowledge scores compared to surgical specialties (beta = 0.91, 95% CI, 0.13 to 1.7, p = 0.024).

Conclusion

This study has revealed a significant lack of knowledge about narcolepsy among physicians in Makkah region. This raises concerns about the timely identification, proper understanding, and accurate diagnosis of patients with narcolepsy. Adequate understanding of narcolepsy is crucial to avoid its misdiagnosis or delays in receiving appropriate treatment and support, ultimately impacting their quality of life.

## Introduction

Narcolepsy is a chronic sleep disorder that affects the central nervous system (CNS). The characteristic of narcolepsy is excessive daytime sleepiness (EDS), and most people experience cataplexy. Cataplexy is described as a transient muscle weakness that starts in the face and neck and then descends to involve the muscles of the trunk and limbs with the preservation of consciousness [1]. It is reported to be triggered by strong, happy emotions such as laughter and excitement. Patients with narcolepsy can also have frequent sleep paralysis and hypnagogic hallucinations [2]. Narcolepsy is classified into two types: type 1 (NT1) and type 2 (NT2). NT1 is distinguished from NT2 by the low level of orexin and the presence of cataplexy, whereas NT2 is known to have a normal level of orexin and no cataplexy [3]. Orexin neurons in the lateral hypothalamus are critical for regulating and maintaining awake. Loss of orexin-producing neurons in persons with narcolepsy causes devastating EDS, sleep episodes, and wake instability [4].

Although narcolepsy is a rare condition, a population-based study over a 10-year period found the incidence rate was increasing from a range of 20.5 to 66.3 per 100,000 person-years, with a predominance in females [5]. Moreover, according to a study done in Europe that compared the incidence of narcolepsy before, during, and after the influenza A (H1N1) pdm09 vaccine campaign, there was a significant increase in the diagnosis of narcolepsy in the 5- to 19-year-old age group in Finland and Sweden [6].

Many cases of narcolepsy appear during adolescence and influence society and the economy due to higher utilization of health care resources, decreased patient function, quality of life, and productivity, negative effects on the patient’s partner and family, and lower employment rates, which lead to lower income [7,8].

Several studies have shown a significant delay in the diagnosis of narcolepsy. A study reported an average of up to 15 years between the beginning of narcolepsy symptoms and the diagnosis. The delay in diagnosis, which has a huge impact on patient life, is likely a result of the lack of awareness of the disease and the recognition of the symptoms by the physician [9]. According to a study done in the United States to evaluate physicians’ knowledge of narcolepsy, only 42% of 100 sleep medicine specialists and 9% of 300 primary care physicians felt “very” or “extremely” comfortable diagnosing narcolepsy. The result demonstrates the lack of awareness and recognition of symptoms among physicians [10].

Increased awareness and early recognition of narcolepsy symptoms among healthcare providers are crucial for helping this population of patients manage their burdensome condition [11]. Narcolepsy has been less well studied among physicians in Saudi Arabia; therefore, this study aimed to assess the knowledge of narcolepsy among physicians in Makkah region of Saudi Arabia.

## Materials and methods

Study design

This study is a web-based, cross-sectional study. Data were obtained from February to November 2023 through an online questionnaire directed to physicians in Makkah region of Saudi Arabia.

Study population (inclusion criteria)

This study targeted all practicing physicians, including medical and surgical specialists, dentists, and physiotherapists of both genders, working in governmental and private healthcare facilities in Makkah region.

Sample size calculation

The minimum sample size required for this study was calculated by Open Epi version 7.0 in consideration of the following: The total number of physicians in Makkah is 12,012 inhabitants (as reported in the last statistics for the year 2017), keeping the confidence interval (CI) level at 95%, considering the percentage of good knowledge about narcolepsy among physicians is between 7% and 22% [10], and taking the design effect as 1. The sample size was calculated to be 226 participants.

Data collection

The study researchers developed a self-administered questionnaire following a review of the literature [10,12]. It was created as a Google form and distributed to the target population through social media platforms such as WhatsApp and Twitter. Participants received a link to the questionnaire that contained an invitation letter containing the inclusion criteria, objectives of the study, and a request to participate willingly. The questionnaire was estimated to require three to five minutes for completion and encompassed several sections, including a consent form, items aimed at gathering demographic and professional data about the participants, as well as items intended to assess their knowledge of narcolepsy. A pilot study was carried out in January 2023 and targeted 22 respondents. The reliability of questionnaire items was evaluated by determining the value of Cronbach’s alpha coefficient, which was 0.82. All responses that were obtained from the pilot study were not included in the main study findings.

Ethical considerations

Ethical approval was obtained from the Umm Al-Qura University Institutional Research Board (IRB) with IRB number HAPO-02-K-012-2023-02-1445. The questionnaire contained an explanation of the purpose and nature of the study as well as a request to participate willingly. Confidentiality of all participants was kept as names, numbers, and sensitive personal information were not included in the questionnaire.

Statistical analysis

The study data was statistically analyzed using RStudio (version 4.3.1). Statistical differences between participants with knowledge levels about narcolepsy were assessed using Pearson’s chi-squared test or Fisher’s exact test. Participants’ knowledge about narcolepsy was based on 12 items. Correct answers were assigned one, and incorrect answers were assigned zero. Therefore, the total score ranged between 0 and 12, and a higher score indicated a higher knowledge level. Factors associated with knowledge were investigated using a univariable linear regression analysis. Predictors of knowledge were then investigated by incorporating the significantly associated variables from the univariable analysis into a multivariable model. Regression analysis results were expressed using beta coefficients and 95% confidence intervals (95% CIs). Statistical significance was considered at p < 0.05.

## Results

Demographic and occupational characteristics

Initially, we collected data from 233 respondents, of whom one declined to participate and six resided outside Makkah region and were excluded. Therefore, data were analyzed from 226 participants. Male physicians constituted 54.4% (n = 123) of the sample. A substantial proportion of the physicians are practicing in governmental hospitals (77.9%, n = 176), and less than half of them reside in Makkah city. Non-surgical specialties represented 73.5% (n = 166). Residents constituted 61.9% (n = 140) of the sample, whereas 33.2% (n = 75) of participants had one to 5 years of experience (Table 1).

**Table 1:** Demographic and occupational characteristics of the study sample

Characteristics	N=226
Gender	
Male	123 (54.4%)
Female	103 (45.6%)
Facility Type	
Governmental hospital	176 (77.9%)
Primary care center	25 (11.1%)
Private hospital	25 (11.1%)
City	
Al-Qunfudah	32 (14.2%)
Jeddah	64 (28.3%)
Makkah	98 (43.4%)
Taif	32 (14.2%)
Specialty	
Surgical	55 (24.3%)
Medical	166 (73.5%)
Dentistry	2 (0.9%)
Physiotherapy/Rehab	3 (1.3%)
Professional Status	
Consultant	41 (18.1%)
Specialist	45 (19.9%)
Resident	140 (61.9%)
Years of Practice	
Less than 1 year	68 (30.1%)
1 to 5 years	75 (33.2%)
5 to 10 years	33 (14.6%)
More than 10 years	50 (22.1%)

Participants’ knowledge regarding narcolepsy and cataplexy and their associated factors

Among respondents aware of narcolepsy, a statistically significant difference was found in the distribution of current levels (p = 0.045). Specifically, a higher proportion of consultants (87.8%, n = 36) and specialists (91.1%, n = 41) reported awareness compared to residents (76.4%, n = 107). There were no significant differences between aware and non-aware participants about narcolepsy in terms of gender, facility type, city, specialty, and years of practice (Table 2).

**Table 2:** Factors associated with the awareness of narcolepsy P: Pearson X^2^ test; $: Fisher’s exact test; *P < 0.05 refers to a statistically significant difference

Characteristic	Aware about narcolepsy		p-value
	No (N=42)	Yes (N=184)	
Gender			
Male	20 (16.3%)	103 (83.7%)	0.326
Female	22 (21.4%)	81 (78.6%)	
Facility Type			
Governmental hospital	33 (18.8%)	143 (81.3%)	0.196^$^
Primary care center	7 (28.0%)	18 (72.0%)	
Private hospital	2 (8.0%)	23 (92.0%)	
City			
Al-Qunfudah	7 (21.9%)	25 (78.1%)	0.614
Jeddah	9 (14.1%)	55 (85.9%)	
Makkah	21 (21.4%)	77 (78.6%)	
Taif	5 (15.6%)	27 (84.4%)	
Specialty			
Surgical	14 (25.5%)	41 (74.5%)	0.189^$^
Medical	27 (16.3%)	139 (83.7%)	
Dentistry	1 (50.0%)	1 (50.0%)	
Physical medicine/Rehab	0 (0.0%)	3 (100.0%)	
Professional Status			
Consultant	5 (12.2%)	36 (87.8%)	0.045^*$^
Specialist	4 (8.9%)	41 (91.1%)	
Resident	33 (23.6%)	107 (76.4%)	
Years of Practice			
Less than 1 year	15 (22.1%)	53 (77.9%)	0.255
1 to 5 years	17 (22.7%)	58 (77.3%)	
5 to 10 years	5 (15.2%)	28 (84.8%)	
More than 10 years	5 (10.0%)	45 (90.0%)	

A total of 123 participants were aware of cataplexy (54.4%). No variables demonstrated significant associations with awareness of cataplexy (p > 0.05) (Table 3).

**Table 3:** Factors associated with the awareness of cataplexy P: Pearson X^2^ test; $: Fisher’s exact test

Characteristics	Aware about cataplexy		p-value
	No (n=103)	Yes (n=123)	
Gender			
Male	49 (39.8%)	74 (60.2%)	0.058
Female	54 (52.4%)	49 (47.6%)	
Facility Type			
Governmental hospital	84 (47.7%)	92 (52.3%)	0.331
Primary care center	11 (44.0%)	14 (56.0%)	
Private hospital	8 (32.0%)	17 (68.0%)	
City			
Al-Qunfudhah	13 (40.6%)	19 (59.4%)	0.902
Jeddah	31 (48.4%)	33 (51.6%)	
Makkah	45 (45.9%)	53 (54.1%)	
Taif	14 (43.8%)	18 (56.3%)	
Specialty			
Surgical	28 (50.9%)	27 (49.1%)	0.807^$^
Medical	73 (44.0%)	93 (56.0%)	
Dentistry	1 (50.0%)	1 (50.0%)	
Physical medicine/Rehab	1 (33.3%)	2 (66.7%)	
Professional Status			
Consultant	20 (48.8%)	21 (51.2%)	0.494
Specialist	17 (37.8%)	28 (62.2%)	
Resident	66 (47.1%)	74 (52.9%)	
Years of Practice			
Less than 1 year	36 (52.9%)	32 (47.1%)	0.508
1 to 5 years	33 (44.0%)	42 (56.0%)	
5 to 10 years	14 (42.4%)	19 (57.6%)	
More than 10 years	20 (40.0%)	30 (60.0%)	

Participants’ responses regarding their knowledge about narcolepsy and cataplexy

Regarding the participants’ knowledge about narcolepsy and cataplexy, several key findings emerged. First, a majority of physicians correctly identified narcolepsy as a sleep disorder (78.3%, n = 177). Regarding the age of onset, about one-third of participants correctly indicated that narcolepsy usually starts in the age group of 10-20 years old (32.3%, n = 73), and a similar proportion (32.3%, n = 73) correctly stated that there are two types of narcolepsy. Furthermore, a significant proportion correctly attributed narcolepsy to multiple causes, including genetic factors, brain injury, and autoimmune disease (32.3%, n = 73). Additionally, a majority correctly identified EDS as the most common symptom of narcolepsy (63.7%). In terms of cataplexy, 40.7% (n = 92) correctly associated it with sudden muscle weakness. When it comes to the diagnosis of narcolepsy, 51.3% (n = 116) correctly identified the multiple sleep latency test (MSLT). Notably, a significant percentage recognized that narcolepsy is not a common disease (69.5%, n = 157), and it can be treated with a combination of behavioral and pharmacologic approaches (81.0%, n = 183). Importantly, less than half of respondents correctly acknowledged that narcolepsy cannot be prevented (41.2%, n = 93), and the majority of them identified that motor vehicle accidents are one of its complications (76.5%, n = 173). Additionally, a considerable proportion acknowledged that there is no cure for narcolepsy (46.9%, n = 106) (Table 4).

**Table 4:** Participants’ responses regarding their knowledge about narcolepsy and cataplexy *An asterisk indicates a correct response

Characteristics	N=226
Narcolepsy is a	
Sleep disorder*	177 (78.3%)
Mental illness	13 (5.8%)
Form of epilepsy	12 (5.3%)
Do not know	24 (10.6%)
Narcolepsy usually starts at	
10-20 years old*	73 (32.3%)
30-40 years old	58 (25.7%)
>50-year-old	10 (4.4%)
Do not know	85 (37.6%)
Narcolepsy caused by	
Genetic	30 (13.3%)
Brain injury	23 (10.2%)
Autoimmune disease	34 (15.0%)
All the above*	73 (32.3%)
Do not know	66 (29.2%)
Types of narcolepsy	
One	3 (1.3%)
Two*	73 (32.3%)
Three	17 (7.5%)
Do not know	133 (58.8%)
The most common symptom of narcolepsy is	
Excessive daytime sleepiness*	144 (63.7%)
Fatigue	22 (9.7%)
Snoring	6 (2.7%)
Do not know	54 (23.9%)
Cataplexy is	
Sudden muscle weakness*	92 (40.7%)
Muscle rigidity, shivering	39 (17.3%)
Do not know	95 (42.0%)
Diagnosis of narcolepsy by	
Multiple sleep latency test*	116 (51.3%)
Hormonal test	8 (3.5%)
Magnetic resonance imaging (MRI)	16 (7.1%)
Do not know	86 (38.1%)
Narcolepsy is a common disease	
False*	157 (69.5%)
True	69 (30.5%)
Narcolepsy is treated with a combination of behavioral and pharmacologic approaches	
False	43 (19.0%)
True*	183 (81.0%)
Narcolepsy could be prevented	
False*	93 (41.2%)
True	133 (58.8%)
Motor vehicle accidents are one of the complications of narcolepsy	
False	53 (23.5%)
True*	173 (76.5%)
There is no cure for narcolepsy	
False	120 (53.1%)
True*	106 (46.9%)

Participants’ responses regarding the diagnostic criteria, prevention of narcolepsy, and common narcolepsy misdiagnosis

The majority of participants (52.2%, n = 118) indicated a lack of knowledge about the diagnostic criteria for narcolepsy in the DSM-5. Among those who attempted to identify the correct diagnostic criteria, 27.4% (n = 62) chose “recurrent periods of an irrepressible need to sleep, lapsing into sleep, or napping occurring within the same day. These must have been occurring at least three times per week over the past three months + episodes of cataplexy, occurring at least a few times per month.” Regarding the prognosis of narcolepsy, 47.8% (n = 108) responded with “I do not know.” For lifestyle modifications, the most commonly selected response was “all of the above” (30.5%, n = 69), encompassing intermittent sleep, nicotine restraint, and taking naps. More than half of the participants did not know about the characteristics associated with narcolepsy type 1. In terms of common narcolepsy misdiagnosis, insomnia was the most frequently chosen response (41.6%, n = 94) (Table 5).

**Table 5:** Participants’ responses regarding the diagnostic criteria, prevention of narcolepsy and common narcolepsy misdiagnosis DSM-5: Diagnostic and Statistical Manual of Mental Disorders fifth Edition *Descriptive data are based on a multiple-response item.

Characteristics	N=226
The diagnostic criteria for narcolepsy according to DSM-5	
Predominant complaint of dissatisfaction with sleep quantity or quality, associated with difficulty maintaining sleep, characterized by frequent awakening or problems returning to sleep after awakening. These must have been occurring at least three times per week over the past three months.	28 (12.4%)
Recurrent periods of an irrepressible need to sleep, lapsing into sleep, or napping occurring within the same day. These must have been occurring at least three times per week over the past three months plus episodes of cataplexy, occurring at least a few times per month	62 (27.4%)
Excessive daytime sleepiness on most days plus habitual loud snoring plus witness apnea or gasping. These must have been occurring at least three times per week over the past three months.	18 (8.0%)
Do not know	118 (52.2%)
Prognosis of the narcolepsy disease	
Good	52 (23.0%)
Bad	66 (29.2%)
Do not know	108 (47.8%)
Lifestyle modifications that should be followed for narcolepsy	
Intermittent sleep	26 (11.5%)
Restrain nicotine	10 (4.4%)
Take naps	33 (14.6%)
All the above	69 (30.5%)
Do not know	88 (38.9%)
Narcolepsy type 1 is characterized by	
Low levels of serotonin	12 (4.9%)
Low levels of chemical hypocretin	52 (23.1%)
High levels of chemical hypocretin	28 (12.4%)
Do not know	134 (59.6%)
Common narcolepsy misdiagnosis*	
Epilepsy/Seizures	65 (28.8%)
Insomnia	94 (41.6%)
Idiopathic hypersomnia	63 (27.9%)
Anxiety/Depression	78 (34.5%)
Obstructive sleep apnea	66 (29.2%)
Stroke	24 (10.6%)

Factors associated with higher participants’ knowledge regarding narcolepsy and cataplexy

In the regression analysis, only participants’ specialty showed a significant association with knowledge regarding narcolepsy and cataplexy. More specifically, participants in medical specialties showed significantly higher knowledge scores compared to those in surgical specialties (beta = 0.91, 95% CI, 0.13 to 1.7, p = 0.024). No other characteristics were associated with participants’ knowledge (Table 6).

**Table 6:** Factors associated with participants’ knowledge regarding narcolepsy and cataplexy 95% CI: 95% confidence interval; *P < 0.05 refers to a statistically significant difference

Characteristics	Beta	95% CI	p-value
Gender			
Male	Reference	Reference	
Female	0.01	-0.67, 0.70	0.976
Facility type			
Governmental hospital	Reference	Reference	
Primary care center	0.74	-0.35, 1.8	0.187
Private hospital	0.34	-0.75, 1.4	0.544
City			
Al-Qunfudah	Reference	Reference	
Jeddah	-0.80	-1.9, 0.31	0.160
Makkah	-0.65	-1.7, 0.40	0.225
Taif	-1.1	-2.4, 0.15	0.086
Specialty			
Surgical	Reference	Reference	
Medical	0.91	0.13, 1.7	0.024^*^
Dentistry	-2.8	-6.5, 0.81	0.130
Physiotherapy/Rehab	-0.15	-3.1, 2.8	0.921
Current level			
Consultant	Reference	Reference	
Specialist	-0.52	-1.6, 0.58	0.353
Resident	-0.55	-1.5, 0.36	0.241
Years of practice			
Less than 1 year	Reference	Reference	
1 to 5 years	0.02	-0.84, 0.87	0.972
5 to 10 years	0.25	-0.84, 1.3	0.652
More than 10 years	0.70	-0.26, 1.6	0.154

## Discussion

Although narcolepsy is considered a rare disease with a prevalence rate of 0.02-0.07% worldwide, there is still a major obstacle in diagnosing this disease and its subsequent negative effects on its prognosis. So, it is of great importance to assess the physician’s awareness of narcolepsy and how they are knowledgeable and capable of early detection of the disease’s manifestations, as well as the diagnostic methods to reduce the significant delay in reaching the diagnosis. A local study of 47 Saudi patients who were diagnosed with narcolepsy showed an average time interval between symptom onset and diagnosis of 8.4 ± 1.2 years. It was also noted that the individuals with the least amount of delay had the youngest age at diagnosis, while the individuals with the most delay had the oldest age at diagnosis [9, 13]. The burden of this delay in diagnosis has a huge impact on various socio-economic factors which have been demonstrated in previous studies. This includes higher utilization of health care resources, decreased quality of life, patient function, and productivity. Moreover, it leads to lower employment rates and income, as well as negative effects on the patient’s partner and family [7]. Furthermore, it has been shown that patients with narcolepsy are more susceptible to psychological disorders and immune diseases [14, 15].

When it comes to the diagnostic methods for narcolepsy, the MSLT is a valuable and most commonly used objective test for sleepiness measurement. It has been shown to be reliable, and it is part of the diagnostic criteria for narcolepsy [16]. In this study, 81.4% (n = 184) of the participants were aware of the term narcolepsy in general, and more than half of them (51.3%, n = 116) were aware of MSLT for the diagnosis of narcolepsy, but the majority of them (52.2%, n = 118) could not know about the diagnostic criteria for narcolepsy in the DSM-5. This certainly supports the fact of inadequate awareness and knowledge among physicians regarding the disease and its valid tools for diagnosis because the DSM-5 criteria are one of the modern and effective methods that help practitioners, especially those who are non-sleep specialists, in diagnosing narcolepsy, as they are simpler, less burdensome, and more practical in clinical practice because they detect the disease based on the presence of EDS plus the fulfillment of at least one of three non-overlapping criteria; in addition, DMS-5 definition of narcolepsy encourages clinicians to gather more detailed information about its likely occurrence, especially in those with recent onset or ambiguous cataplexy [12].

The current results showed no significant difference between aware and non-aware participants about narcolepsy in terms of specialty, which is interestingly compatible with another result of a similar study applied in Croatia that also included physicians in different specialties [17]. Physicians in medical (non-surgical) specialties were predicted to have higher knowledge scores about narcolepsy and cataplexy compared to the surgical specialties, which is consistent with another study in the United States which reported that sleep medicine specialists (neurologists, psychiatrists, and pulmonologists) were evidently more knowledgeable in comparison with the primary care physicians [10]. Perhaps one of the reasons behind this is that narcolepsy is closely linked to neuropsychiatric disorders; hence, the presence of manifestations related to these disorders may constitute a major challenge in reaching the diagnosis of narcolepsy and diagnosing patients instead with other psychological diseases such as depression and anxiety [17].

In a previous study aimed at assessing the journey of narcolepsy patients with the diagnosis, it was found that 60% of patients had been diagnosed with other illnesses prior to narcolepsy, and the vast majority of them had a primary diagnosis of depression. There are also many different studies that have shown similar results, showing that what most patients are diagnosed with before narcolepsy is usually a mental illness [18]. All these findings intensify the importance of increasing physicians’ awareness regarding narcolepsy.

Strengths and limitations

The study is considered a valuable source of evidence due to the small number of Saudi studies published in this field. The findings of this study can help inform future policy and research in Saudi Arabia and provide insight into the current situation. It can also help to identify areas for improvement and create strategies for better outcomes. In addition to having participants with varying specialties and professional statuses, this study has the advantage of including a diverse group of participants. As a result, the authorities could deal with the issue from all angles. This study also provides a solid foundation for further investigation as well as laying the groundwork for future research.

The study has several limitations. As the sample size was small, the results could not be generalized to Saudi Arabian physicians. The statistical significance would also have been improved by increasing the sample size. A final limitation is that the study only examined one region within Saudi Arabia, so results may differ based on regional differences.

## Conclusions

Physicians’ knowledge of narcolepsy is inadequate in Makkah region; the medical specialty is the sole factor that affects the knowledge level of this disorder among physicians. Frequent studies are recommended in this area across different regions and specialties. Also, to increase awareness among physicians about narcolepsy, this could be achieved through continuous medical education programs, seminars, and workshops. Additionally, providing resources, such as books and journals, would help spread knowledge about narcolepsy. Finally, encouraging research and clinical studies on narcolepsy would help to further our understanding of the condition.
